# A Mini Review on Antidiabetic Properties of Fermented Foods

**DOI:** 10.3390/nu10121973

**Published:** 2018-12-13

**Authors:** Bhagavathi Sundaram Sivamaruthi, Periyanaina Kesika, Mani Iyer Prasanth, Chaiyavat Chaiyasut

**Affiliations:** 1 Innovation Center for Holistic Health, Nutraceuticals, and Cosmeceuticals, Faculty of Pharmacy, Chiang Mai University, Chiang Mai 50200, Thailand; p.kesika@gmail.com; 2 Age-Related Inflammation and Degeneration Research Unit, Department of Clinical Chemistry, Faculty of Allied Health Sciences, Chulalongkorn University, Bangkok 10330, Thailand; prasanth.m.iyer@gmail.com

**Keywords:** fermented foods, diabetes, alternative medicine, fermentation

## Abstract

In general, fermented foods (FFs) are considered as functional foods. Since the awareness about the health benefits of FFs has increased, the consumption of FF also improved significantly in recent decades. Diabetes is one of the leading threats of the health span of an individual. The present manuscript details the general methods of the production of FFs, and the results of various studies (in vitro, in vivo, and clinical studies) on the antidiabetic properties of FFs. The fermentation method and the active microbes involved in the process play a crucial role in the functional properties of FFs. Several in vitro and in vivo studies have been reported on the health-promoting properties of FFs, such as anti-inflammation, anticancer, antioxidant properties, improved cognitive function and gastrointestinal health, and the reduced presence of metabolic disorders. The studies on the functional properties of FFs by randomized controlled clinical trials using human volunteers are very limited for several reasons, including ethical reasons, safety concerns, approval from the government, etc. Several scientific teams are working on the development of complementary and alternative medicines to improve the treatment strategies for hyperglycemia.

## 1. Introduction

Fermented foods (FFs) are the product of the microbial conversion of raw food materials. FFs have been prepared and used for several thousand years, perhaps since the development of civilization. Almost all humans are exposed to FFs in many forms such as dairy products, vegetables (pickled), meat, and fish products. The use and types of FFs vary among the people based on the geographical region, climatic conditions, and availability of the source. The preparation methods greatly influence the quality of the FFs. The microbes involved in the fermentation process determines the type and quality of the FFs. Lactic acid bacteria (LAB), Propionibacterium, Acetobacter, yeast, molds, and *Bacillus* species are the common microbes involved in the fermentation process, and are responsible for the presence of lactic, acetic, and propionic acids, alcohol, ammonia, and fatty acids [1]. The fermentation process improves the quality (nutritional and organoleptic) and health-promoting potential of foods, as well as extends their shelf life [2,3,4]. 

The microorganisms that naturally occur in the raw materials are responsible for the fermentation process of traditional fermented foods. To avoid the contamination of pathogenic microbes, a harmful by-product produced by unwanted microbes during the fermentation, and obtain an enhanced health beneficial fermented product, the fermentation process has been modernized by developing appropriate steps such as starter culture production, controlled multi-step fermentation, and fermented functional foods production. Therefore, identification of the naturally occurring microbes is necessary for the preparation of the starter culture [5]. Starter cultures are chosen based on the quality, safety, and shelf life of the respective fermented product. Then, the environmental conditions are optimized to obtain a better performance of the starter culture. The strains of starter cultures within the species are evaluated to obtain a suitable starter culture with high efficiency or a unique property of the respective fermented product. Even for a controlled multi-step fermentation process, the prepared cultures are used to obtain a better quality of the fermented product [5].

The FFs have been reported as having several health benefits such as the prevention and managing metabolic disorders, cardiovascular diseases, cognitive improvement, immune enhancement, etc. [6,7,8,9,10,11,12]. The FFs prepared with beneficial microbes help improve the health status of consumers. For example, the ingestion of galactosidases producing microbes via FFs help people consume dairy products without any consequences of lactose intolerance, [2] and it has been reviewed that FFs are a multisource of bioactive microbes with multiple health-promoting properties [13]. The fermented plant extracts have also been used for the development of cosmetic products [14]. However, the proposed findings must be proven by clinical trials to confirm the functionality of the food. Recent scientific reports have proposed that the supplementation of fermented foods diminish the diabetes mellitus (DM)-associated health consequences [15,16,17]. The current review provides the cumulative information about the recent reports on the antidiabetic properties of fermented foods, and discusses the sustainable use of functional fermented foods for the efficient management of the DM condition.

## 2. Antidiabetic Properties of Fermented Foods

### 2.1. In Vitro Studies 

Soymilk was fermented with Kefir culture (*Lactobacillus plantarum*, *L. casei*, *Leuconostoc cremoris*, *Streptococcus lactis*, *S. cremoris*, *S. diacetylactis*, and *Saccharomyces fragilis*) and Rhodiola crenulata extracts, and the antidiabetic property of the phenolic extracts of the fermented soymilk was assessed via enzyme assays (α-amylase, α-glucosidase, and angiotensin-converting enzyme (ACE) inhibitory activities) [18]. The fermentation increased the content of tyrosol, while it decreased the salidroside contents and improved the α-glucosidase-inhibiting ability. The reduction in α-amylase inhibition activity also contributes beneficial effects by possessing fewer side effects such as flatulence, abdominal distension, etc. Further, ACE inhibitory activity was found to be altered when compared to non-fermented soymilk. The results suggested that the fermentation process improved the quality of soymilk, which can be used to the treat type 2 diabetic condition [18] (Table 1).

About 60% of 2-[N-(7-nitrobenz-2-oxa-1,3-diazol-4-yl) amino]-2-deoxy-D-glucose uptake was found to be observed in C2C12 cells when they were exposed to 50 μg/mL ethanol extract of the fermented rice bran and soybean (FRBSB). The FRBSB exposure increased the phosphorylation of Akt in a dose-dependent manner, and also promoted the insulin signal transmission via phosphorylating the glucose synthase kinase-3β. The FRBSB exposure does not affect the AMPK (5’ adenosine monophosphate-activated protein kinase) signaling. The results suggested that the FRBSB improved the glucose uptake via the PI3 kinase/Akt pathways [19].

Rice wine (Makgeolli) prepared with various concentrations of *Laminaria japonica* J.E. Areschoug has been studied for its alcohol, sugar contents, viable cell count, and protein tyrosine phosphatase 1B inhibition properties. The varying concentrations of *L. japonica* has not significantly affected the sugar, alcohol, and microbial content. The presence of 5–7.5% of *L. japonica* in Makgeolli has been accepted by human volunteers. The Makgeolli prepared with 5–7.5% of *L. japonica* exhibited a considerable level of protein tyrosine phosphatase 1B inhibitory activity, and overall acceptability [20]. 

The platelets derived from type 2 diabetic patients were treated with fermented papaya preparation, and the enzyme activity and antioxidant capacity was assessed in vitro conditions. Fermented papaya preparation treatment improved the membrane fluidity, Na^+^/K^+^-ATPase activity, total antioxidant capacity, and superoxide dismutase activity, and greatly reduced the conjugated diene levels in platelets when compared with the control. The findings proved that the fermented papaya treatment improved the function of platelets, and protects the system from oxidative stress [21].

*Myriciaria dubia* Mc. Vaugh, commonly known as camu-camu, has been fermented using LAB strains (*L. plantarum*, and *L. helveticus*) along with soymilk for 72 h, and the samples were periodically collected and subjected to several phytochemical analysis and enzyme assays. The α-amylase and α-glucosidase inhibitory actions were significantly increased in the fermented product, while no predominant changes were observed in antioxidant property and total phenolic content. ACE-inhibiting ability was increased in *L. plantarum*-mediated fermented camu-camu in the presence of soymilk. The study claimed that the LAB-mediated fermented camu-camu can be considered a nutraceutical supplement to manage type 2 diabetes [22].

### 2.2. In Vivo Studies in Animal Models

*L. acidophilus*, *Streptococcus thermophillus*, and *B. longum* strains were used to ferment milk, which contain aqueous extract of four mushrooms (*Lentinus edodes* (Berk.) Singer, *Flammulina velutipes* (Curtis) Singer, *Ganoderma lucidum* (Curtis) P. Karst, and *Pleurotus ostreatus* (Jacq. ex Fr.) P. Kumm.). Streptozotocin (STZ)-induced diabetic Otsuka Long-Evans Tokushima fatty (OLETF) rats were fed 10–20% fermented milk for six weeks, and the changes in their weight, fat, and lipid profile were measured. The results showed that the supplementation of fermented milk effectively reduced the weight, perirenal, visceral and epididymal fats, serum triglyceride, and non-esterified fatty acid content in OLETF rats when compared to control. The lipid profile was not significantly changed during the intervention [23]. The supplementation of fermented mycelia of *Cordyceps sinensis* (Berk.) Sacc., fruiting body, and fermented broth to diabetic rat for two weeks, in the concentration of one grams per day, significantly reduced blood glucose and fructosamine levels [24] (Table 2).

OLETF rats with type 2 DM were fed with fermented grain foods known as Antioxidant Biofactor (AOB). About 6.5% of AOB was supplemented (for four weeks) via feed to type 2 DM rats. The supplementation of AOB effectively reduced the blood glucose level, hemoglobin A1c (HbA1c), serum triglyceride, plasminogen activator inhibitor-1, cholesterol, and low-density lipoprotein (LDL), in OLETF rats. Moreover, AOB intervention normalized the expression of UCP2 (one of the regulators of reactive oxygen species (ROS) production) and increased the endothelial nitric oxide synthase (eNOS) proteins, which is associated with the nitric oxide–cyclic guanosine monophosphate (NO-cGMP) pathway. Collectively, the results revealed that the supplementation of AOB improved the health status of diabetic rats via the NO-cGMP pathway [25]. 

The supplementation of fermented rice bran and soybean (0.4% in diet) for 10 weeks significantly reduced the glucose, HbA1c, and serum triglyceride in KK-A^y^/Ta Jc mice. The antidiabetic nature of the fermented product has been attributed to the increased glucose uptake, which has been proved using C2C12 cells. The exposure of ethanol extract of fermented rice bran and soybean increased the uptake of 2-NBDG via PI3kinase/Akt pathways [19]. 

*Monascus purpureus* Went (Monascaceae) NTU 568 is commonly known as red mold and corn silage mold, which was used to ferment the dioscorea root, long-grain rice, and adlay. STZ-induced diabetic Wistar rats were fed with the fermented products for eight weeks. After the intervention period, rats were assessed for improvements in diabetic-related discomforts. The results proved that the supplementation of red mold fermented products meritoriously reduced the plasma glucose, triglyceride, amylase, and cholesterol levels, and ROS production. A notable increase in the activities of glutathione disulfide reductase, glutathione reductase, and catalase was observed in the diabetic rats fed with red mold fermented products [26]. 

The aqueous extract of short fermented tea (co-fermented with loquat leaf and green tea leaf) extract reduced the maltose-induced blood glucose rise in Sprague–Dawley rats, but there was no impact when sucrose or glucose was administered to the rats. The results suggested that the supplementation of fermented tea inhibited the maltase activity [27]. 

Yeast fermented aged black garlic product (YFBG) was supplemented to high-fat diet induced obese and diabetic ICR (Institute of Cancer Research) mice for nine weeks at various concentrations. In all of the concentrations, YFBG supplementation showed protective effects in obese mice. The intervention of YFBG reduced the body mass, adipocyte diameters, periovarian fat weight, abdominal fat, blood glucose, aspartate aminotransferase (AST), alanine aminotransferase (ALT), blood urea nitrogen (BUN), and creatinine levels, and rectified the damages in kidney tubules. The study was compared with aged black garlic, it has been revealed that the fermentation process improved the protective property of aged black garlic, significantly [28]. 

Fermented legume product (Bambara groundnut, African locust bean, and soybean) was supplemented to STZ-induced diabetic Wistar rats for two weeks at various combinations and concentrations. The supplementation reduced the activities of alkaline phosphatase (ALP), ALT, AST, and malondialdehyde while improving the glutathione S-transferase and catalase activities, suggesting that the beneficial effects of the fermented product might be due to the presence of phenolic content and free radical scavenging activity. Glutathione levels were also enhanced after the supplementation of fermented legume products [29]. 

The supplementation of fermented *Morinda citrifolia* L. declined the HbA1c, blood glucose, serum triglyceride, LDL levels, and increased the insulin sensitivity in KK-A^y^/TaJcl mice (type 2 DM) via activation of peroxisome proliferator-activated receptor (PPAR) γ, and AMP-activated protein kinase [30]. 

Fermented soybean (FSB) was prepared using *Bacillus subtilis* MORI, and the antidiabetic property of FSB was assessed using *db*/*db* mice. The mice were randomly divided and were fed with 125 mg per kg or 500 mg per kg of FSB or control diet for eight weeks. After the intervention, animals were evaluated for selected parameters. The results suggested that the supplementation of FSB effectively reduced the HbA1c and blood glucose levels, and improved the plasma insulin content in *db*/*db* mice when compared to control [31]. 

Diabetic (type 2 DM) rats were fed with fermented soybeans (Chungkookjang), and/or Jerusalem artichokes for eight weeks, and the results suggested that the supplementation of both Chungkookjang and fresh Jerusalem artichoke (*Helianthus tuberosus* L.) improved the diabetic-associated health status. The intervention reduced the visceral fat, hepatic glucose, and triglyceride levels and improved the insulin secretion, hepatic insulin sensitivity, and glucose tolerance, and also increased the β-cell mass. Collectively, the study proved that the fermented soybeans and Jerusalem artichokes supplementation enhanced the β-cell function and reversed the insulin resistance in diabetic rats [32].

*Mardi Rhizopus* sp. strain 5351 mediated fermented mung bean (FMB) was supplemented to alloxan-induced diabetic mice in two different concentration (200 mg per kg or 1000 mg per kg of body weight) and the anti-hyperglycemic activity was assessed. The results showed that the supplementation of FMB does not exhibit hypoglycemic effects in the normal rat, whereas FMB supplementation increased the insulin secretion and antioxidant levels while reducing the cholesterol, triglyceride, and LDL content in diabetic mice. The enhanced antidiabetic activity of FMB was attributed to the high content of GABA (γ-aminobutyric acid) and free amino acids [33]. 

The supplementation of fermented *Rhynchosia nulubilis* (Yak- Kong) (500 mg/kg of body weight) for four weeks significantly improved the body mass, high-density lipoprotein (HDL), and glucose tolerance while reducing the serum glucose level, cholesterol, triglyceride, LDL, malondialdehyde, and coronary risk factors (AST and ALT level) in alloxan-induced diabetic rats [34]. Cheonggukjang-mediated fermented green tea (FGT) reduced the HbA1c, glucose levels, and insulin resistance in KK-A^y^ (type 2) diabetic mice. The molecular studies proved that the supplementation of FGT improved the expression of glycolysis-associated genes up to several folds [35]. 

The supplementation of Kombucha, fermented sugared black tea, (six mg per kg of body weight) for 45 days to STZ-induced diabetic rats significantly reduced the HbA1c levels and increased the insulin, hemoglobin, and tissue glycogen levels. Kombucha treatment also improved the functionality of gluconeogenic and glycolytic enzymes in experimental rats. The results suggested that Kombucha has a hypoglycemic effect, which can be used as a functional food supplement to effectively manage the diabetic conditions [15]. The supplementation of fermented green tea with Aquilariae lignum improved the overall health status of type 2 diabetic *db*/*db* mouse by diminishing the consequences of hyperglycemia, hyperlipidemia, and obesity [36].

The supplementation of fermented soy permeates (FSP; 100 mg/day) for three weeks significantly improved the antioxidant status and anti-inflammatory system in STZ-induced diabetic (type 1) rats. The level of carboxymethyl-lysine, a known systemic oxidative stress marker, was reduced, and the activities of superoxide dismutase (SOD) and glutathione peroxidase (GPX) were increased after FSP supplementation in diabetic rats. The expression of manganese superoxide dismutase (Mn-SOD) was increased while the amount of interleukin-1β (IL-1β) and uric acid were reduced in FSP-supplemented diabetic rats. The results revealed that the supplementation of FSP can improve the wellness of diabetic rats by improving the antioxidant and anti-inflammatory system [37]. 

LAB-mediated fermented *Momordica charantia* L. (bitter melon) aqueous extract or ethanol extract protected the alloxan-induced diabetic mice from liver damage and controlled the blood glucose level, significantly than that of the control [38]. 

*Monascus purpureus* 254-mediated fermented rice was supplemented (100 mg/kg or 200 mg/kg of body weight) to STZ-induced diabetic rats, and the antidiabetic effects were assessed. The results showed that fermented rice supplementation significantly reduced the HbA1c, glucose, LDL, very low density lipoprotein (VLDL), cholesterol, triglyceride, urea, creatinine, uric acid, glutathione (GSH), and BUN levels, as well as GPX activities, and increased the body mass, SOD, catalase (CAT), HDL, insulin levels, and total protein content in diabetic rats. The study proved that *M. purpureus*-mediated fermented rice enhanced the health status of diabetic rats by improving the antioxidant system and kidney function [39].

LAB-mediated fermented milk, which contains conjugated linoleic acid (FM), was supplemented (0.2 or 0.6%) to *db*/*db* mice (type 2 DM) for six weeks, and the diabetic-associated parameters were assessed. The supplementation of FM effectively reduced the body weight, fasting blood glucose, cholesterol, and triglyceride levels, and improved the glucose tolerance. The level of LDL and HDL were decreased and increased, respectively, in FM supplemented diabetic mice. The results revealed that the supplementation of FM significantly improved the health status of type 2 diabetic mice [40]. 

The supplementation of *L. plantarum*-fermented purple Jerusalem artichoke hot water extract successfully reduced the blood sugar, cholesterol, triglycerides, and non-essential fatty acids levels, as well as α-glucosidase activity, while improving the insulin and HDL levels in diabetic mice [41]. 

A mixture of the LAB (*Streptococcus thermophilus*, *L. bulgaricus*, *L. acidophilus*, and *B. adolescentis*)-mediated fermented milk with different concentrations of *Anemarrhena asphodeloides* Bunge (FMAA) were prepared, and the biochemical and antidiabetic property was assessed using STZ-induced diabetic mice. FMAA was enriched with viable cells of LABs. The supplementation (10 mL/kg of body weight) of FMAA improved the insulin level and SOD activity, and significantly reduced the common diabetic related parameters such as blood glucose, cholesterol, triglycerides, BUN, LDL, creatinine, and malondialdehyde in experimental mice [42]. 

The supplementation of fermented *Moringa oleifera* Lam. (FMO; 200 mg/kg of body weight) for 10 weeks improved the glucose tolerance and reduced the oxidative stress in high-fat diet-induced obese C57BL/6 mice. The genes involved in liver lipid metabolism, and proinflammatory cytokine expression were up-regulated and down-regulated, respectively in the FMO-supplemented mice. The results suggested that FMO reduced the high-fat diet-induced health defects, effectively by reducing inflammation and oxidative stress [43]. 

*L. fermentum*-mediated fermented skim milk supplementation reduced the blood glucose, cholesterol, LDL, and urea in alloxan-induced diabetic rats when compared to control [44]. The supplementation of fermented food paste (Xeniji™) reduced the blood glucose levels and increased the insulin sensitivity, lipid metabolism, and glucose metabolism in STZ-induced diabetic (type 2) mice. The levels of proinflammatory cytokines (nuclear factor NF-κB and inducible nitric oxide synthase (iNOS)), nitric oxide, cholesterol, triglycerides, AST, ALT, and ALP were reduced in high-fat diet and STZ-induced diabetic mice upon Xeniji™ supplementation [16].

### 2.3. In Vivo Studies in Humans

Prediabetic volunteers were supplemented with fresh or fermented kimchi for 16 weeks (eight weeks of fresh kimchi supplementation—four weeks of washout period—eight weeks of fermented kimchi supplementation; 100 g of kimchi per meal). After the intervention period, the body weight, waist perimeter, and body mass index were found to be significantly reduced in all of the subjects supplemented with fresh and fermented kimchi. The supplementation of fermented kimchi for 10 days effectively reduced the insulin resistance and blood pressure, and improved the insulin sensitivity, quantitative insulin sensitivity check index (QUICKI), and disposition index scores. About 33.3% of volunteers showed positive progress in their health status after the consumption of fermented kimchi. The study suggested that the consumption of an adequate amount of kimchi in a daily meal helps manage the diabetes-related consequences [7] (Table 3).

Hypertensive and type 2 diabetic patients were supplemented with Korean traditional diet (prepared similar to the diet menu of Korean by 1970s) for 12 weeks. The traditional Korean diet is mainly comprised of vegetables and fermented foods. After 12 weeks of a parallel controlled study, a significant level of reduction in HbA1c was observed among the patients who consumed the traditional diet when compared to the control group. Moreover, heart rates were improved during the study period. The results suggested that the consumption of the Korean traditional diet, which is mostly made of fermented foods, improved the quality of hypertensive and type 2 diabetic patients’ lives and reduced the risk of cardiovascular diseases [45].

Type 2 diabetic patients were supplemented with 600 mL of kefir (a probiotic LAB-fermented milk) per day for eight weeks. The kefir supplementation significantly reduced HbA1c levels when compared with baseline and placebo, while no significant changes were observed in triglyceride levels, total cholesterol, LDL, or HDL cholesterol levels. The study stated that kefir can be used as a complementary food supplement to manage diabetes [46]. Another study by Alihosseini et al. [47] also reported that the supplementation 600 mL of kefir per day for eight weeks significantly reduced the homeostatic model assessment of β-cell function and insulin resistance (HOMA-IR) values and homocysteine levels in type 2 diabetic patients, while no prompt changes were observed in serum insulin levels [47].

## 3. Conclusions

**Figure 1:**
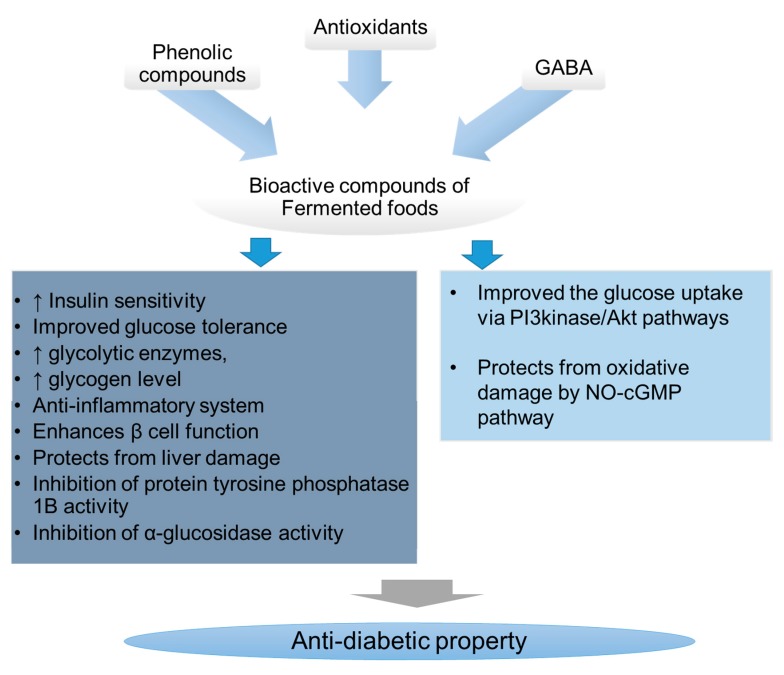
A Schematic representation of possible mechanism of anti-diabetic activity of bioactive compounds of fermented foods. GABA: γ-aminobutyric acid.

So far, the findings of several studies suggested that the supplementation of FFs significantly reduced the diabetic-associated health complications by increasing the antioxidant capacity and anti-inflammatory machinery of the host system. Some of the studies, for example, the supplementation of cascade-fermented dietary supplements containing fruits, nuts, vegetables, chromium, and zinc have not shown any significant health improvement, in terms of HbA1c, fructosamine, and fasting glucose levels, in type 2 diabetic patients [48]. Thus, the study suggests that not all of the fermented products have antidiabetic properties. Several factors such as the fermentation process, the microbial strain involved in the fermentation, and the raw materials used play a critical role in the bioactivity of the finished product. Several studies have reported that the phenolic compounds, antioxidants, and GABA are some of the bioactive compounds that might be responsible for the antidiabetic property of the fermented foods (Figure 1). The complete mechanism of fermented foods on managing the DM is not known. Many in vivo studies have claimed that the respective FFs exhibited significant anti-hyperglycemic activity in rat and mice models. However, the subsequent clinical studies are not enough to prove the findings of in vivo studies. The detailed randomized placebo-controlled clinical trials are necessary to confirm the bioactivity of FFs, which aids in the development and marketing of FF-based alternative remedies for the management of hyperglycemia.

## Figures and Tables

**Table 1:** Reported in vitro studies revealing the antidiabetic properties of fermented foods.

Study Material	Findings	Reference
Fermented soymilk with *Rhodiola crenulata* extracts	↑ α-glucosidase inhibitory activity. ↑ Tyrosol content. ↓ Salidroside content. ↓ α-amylase inhibitory activity Altered the ACE inhibitory activity.	[18]
Ethanol extract of fermented rice bran and soybean	↑ Phosphorylation of Akt ↑ Absorption of 2-NBDG. ↑ Phosphorylation of glucose synthase kinase-3β. No change in AMPK signaling activation	[19]
Rice wine made with *Laminaria japonica* J.E. Areschoug	↑ Protein tyrosine phosphatase 1B inhibition property	[20]
Fermented papaya	↑ Platelet function. ↑ Na^+^/K^+^-ATPase activity. ↑ Membrane fluidity ↑ TAC ↑ SOD activity ↓ Conjugated diene levels	[21]
LAB-mediated fermented Camu-camu and soymilk	↑ α-glucosidase and α-amylase inhibitory activity. Changed ACE inhibitory activity. No significant changes in antioxidant activity and phenolic content.	[22]

Abbreviations: 2-NBDG, A fluorescent deoxyglucose analog; ACE, Angiotensin converting enzyme; Akt, Protein kinase B; AMPK, 5’ adenosine monophosphate-activated protein kinase; SOD, Superoxide dismutase; TAC, Total antioxidant capacity; LAB, Lactic acid bacteria; ↓, Decreased; ↑, Increased.

**Table 2:** Reported in vivo studies in animal models revealing the antidiabetic properties of fermented foods.

Model System	Study Material	Findings	Reference
STZ-induced diabetic rats	Fermented tea beverage	↓ HbA1c ↑ Insulin, hemoglobin and tissue glycogen. Normalized the activities of glucose-6-phosphatase, fructose-1,6-bisphosphatase, and hexokinase	[15]
STZ-induced diabetic mice	Fermented food paste (Xeniji™)	↓ Blood glucose ↓ Leptin ↑ Insulin sensitivity ↑ Lipid and glucose metabolism ↓ Proinflammatory cytokines ↓ NF-κB and iNOS gene expression ↓ Nitric oxide level ↓ Cholesterol ↓ Triglycerides ↓ AST, ALT, ALP ↑ Glycogen level ↓ IL-1β and TNF-α	[16]
KK-A^y^/Ta Jc mice	Fermented rice bran and soybean	↓ HbA1c ↓ Blood glucose ↓ Serum triglyceride ↑ Glucose uptake	[19]
STZ-induced diabetic OLETF rats	Fermented milk with mushroom extract	↓ Body mass ↓ Perirenal, visceral, and epididymal fats ↓ Serum triglyceride ↓ Non-esterified fatty acid	[23]
Nicotinamide and STZ-induced diabetic rats	Fermented *Cordyceps sinensis* (Berk.) Sacc.	↓ Diabetic-associated symptoms	[24]
OLETF rats	Fermented grain foods (Antioxidant biofactor)	↓ HbA1c ↓ Blood glucose ↓ Plasminogen activator inhibitor-1 ↓ Serum triglyceride ↓ LDL, cholesterol Normalized the UCP2 expression ↑ eNOS proteins	[25]
STZ-induced diabetic Wistar rats	Red mold fermented products	↓ Plasma glucose, triglyceride, amylase, and cholesterol levels. ↑ Glutathione disulfide reductase, glutathione reductase, and catalase activity. ↓ ROS	[26]
Sprague-Dawley rats	Fermented tea	↓ Blood glucose level	[27]
ICR mice	Fermented aged black Garlic	↓ Body mass ↓ Adipocyte diameters ↓ Periovarian fat weight ↓ Abdominal fat ↓ Blood glucose level ↓ AST and ALT levels ↓ BUN and creatinine levels Normalized the kidney tubules	[28]
STZ-induced diabetic Wistar rats	Fermented legume condiment	↓ AST, ALT, ALP, and malondialdehyde ↑ Glutathione S-transferase, catalase activity ↑ Glutathione level	[29]
KK-A^y^/TaJcl mice	Fermented noni	↓ HbA1c ↓ Blood glucose ↓ Serum triglyceride, LDL ↑ Insulin sensitivity	[30]
*db*/*db* Mice	Fermented soybean	↓ HbA1c ↓ Blood glucose ↑ Plasma insulin level	[31]
Sprague–Dawley rats	Fermented soybeans (Chungkookjang), and Jerusalem artichoke (*Helianthus tuberosus* L.)	↓ Visceral fat ↑ Glucose tolerance ↑ Insulin secretion ↓ Hepatic glucose ↓ Triglyceride ↑ Insulin sensitivity ↑ β-cell mass	[32]
Alloxan-induced diabetic mice	Fermented mung bean extracts	↓ Cholesterol ↓ Triglyceride ↓ LDL ↑ Insulin secretion ↑ Antioxidant level	[33]
Alloxan-induced diabetic rats	Fermented *Rhynchosia nulubilis* (Yak-Kong)	↑ Body mass ↑ HDL ↑ Glucose tolerance ↓ Glucose level ↓ Cholesterol ↓ Triglyceride ↓ LDL ↓ Coronary risk factors ↓ Malondialdehyde	[34]
KK-A^y^ diabetic mice	Fermented green tea	↓ HbA1c ↓ Glucose level ↓ Insulin resistance ↑ Glycolysis associated gene expressions	[35]
*db*/*db* mouse	Fermented green tea	↓ Body weight ↓ Food and water intake ↑ Fecal excretion ↓ Periovarian fat pad mass ↓ White adipocyte diameters ↓ Depths of deposited fat ↑Adiponectin ↓ Serum leptin levels ↓ Pancreatic weight ↓ Pancreatic islet numbers ↓ Blood glucose levels ↑ Insulin level ↓ LDL ↑ HDL ↓ Triglyceride ↓ Serum AST and ALT levels ↓ Steatohepatitis regions ↓ Hepatocyte hypertrophies ↓ BUN and creatinine levels ↓ Lipid peroxidation ↑ GSH ↑ CAT and SOD activity	[36]
STZ-induced diabetic rats	Fermented soy permeates	↓ Carboxymethyllysine ↑ SOD and GPx activities ↑ Mn-SOD expression ↓ IL-1β ↓ Uric acid	[37]
Alloxan-induced diabetic mice	Fermented *Momordica charantia* L. extract	↓ Triglyceride ↓ Glucose level ↑ HDL	[38]
STZ-induced diabetic rats	*Monascus purpureus* Went (Monascaceae) 254 fermented rice	↓ HbA1c ↓ Glucose level ↑ Body weight ↑ HDL ↓ LDL ↓ VLDL ↓ Cholesterol ↓ Triglyceride ↑ Insulin level ↓ Urea ↓ Creatinine ↓ Uric acid ↓ BUN ↑ Total protein ↑ SOD, CAT, GSH, and GPX activities	[39]
*db*/*db* mice	Fermented milk with conjugated linoleic acid	↓ Body weight ↓ Blood glucose ↑ Insulin, and leptin level Improved the glucose and insulin tolerance. ↓ Cholesterol ↓ Triglycerides ↓ LDL ↑ HDL	[40]
C57BIKsJ *db*/*db* mice	Fermented purple *Jerusalem artichoke* (*Helianthus tuberosus* L.) extract	↓ Blood glucose ↑ Insulin ↑ HDL ↓ Cholesterol ↓ Triglycerides ↓ Non-essential fatty acids ↓ α-glucosidase activity	[41]
STZ-induced diabetic mice	Fermented milk with *Anemarrhena asphodeloides* Bunge	↓ Blood glucose ↓ Food consumption ↓ Cholesterol ↓ Triglycerides ↓ BUN ↓ LDL ↓ Creatinine ↓ Malondialdehyde ↑ Insulin ↑ SOD	[42]
C57BL/6 mice	Fermented *Moringa oleifera* Lam	↑ Glucose tolerance ↓ Glucose intolerance ↓ Lipid accumulation ↑ Lipid metabolism ↓ Lipotoxicity ↓ Oxidative stress ↓ Proinflammatory cytokine ↓ Inflammation	[43]
Alloxan-induced diabetic rats	Fermented milk	↓ Blood glucose ↓ Cholesterol ↓ LDL ↓ Urea	[44]

Abbreviations: NF-κB, Nuclear factor-κB; iNOS, Inducible nitric oxide synthase; IL-1β, Interleukin-1β; TNF-α, Tumor necrosis factor-α; ALP, Alkaline phosphatase; ALT, Alanine transaminase; AST, Aspartate transaminase; BUN, Blood urea nitrogen; ROS, Reactive oxygen species; CAT, Catalase; eNOS, Endothelial nitric oxide synthase; GSH, Reduced glutathione; GPX, Glutathione peroxidase; Mn-SOD, manganese superoxide dismutase; HDL, High-density lipoprotein; LDL, Low-density lipoprotein; OLETF, Otsuka Long-Evans Tokushima fatty; SOD, Superoxide dismutase; STZ, Streptozotocin; TAC, Total antioxidant capacity; VLDL, Very low density lipoprotein; ↓, Decreased; ↑, Increased. UCP2, Mitochondrial uncoupling proteins 2. ICR, Institute of Cancer Research.

**Table 3:** Reported in vivo studies in humans revealing the antidiabetic properties of fermented foods.

Subjects	Study Material	Findings	Reference
Volunteers with prediabetes (*n* = 21)	Kimchi (fresh and fermented)	↓ Body mass, body mass index, and waist perimeter. ↓ Insulin resistance, and blood pressure. ↑ Insulin sensitivity, QUICKI and disposition index.	[7]
Hypertensive and type 2 diabetic patients (*n* = 41)	Korean traditional foods (vegetable and fermented foods)	↓ HbA1c Improved the heart rate	[45]
Type 2 diabetic patients (*n* = 30)	Kefir (probiotic fermented milk)	↓ HbA1c No significant changes in serum triglyceride, total cholesterol levels.	[46]
Type 2 diabetic patients (*n* = 60)	Kefir (probiotic fermented milk)	No changes in serum insulin level. ↓ HOMA-IR value ↓ Homocysteine level ↑ QUICKI	[47]

Abbreviations: HbA1c, Hemoglobin A1c; HOMA-IR, Homeostatic model assessment of β-cell function and insulin resistance; QUICKI, Quantitative insulin sensitivity check index; ↓, Decreased; ↑, Increased.
